# Primary liposarcoma of the omentum: A case report

**DOI:** 10.1097/MD.0000000000028344

**Published:** 2022-01-07

**Authors:** Ying Gao, Yujie Qin, Yingchao Wang, Xiaoling Quan, Xiaoyi Wei, Jiaxi Yao

**Affiliations:** a Department of Obstetrics and Gynecology, Hexi University Affiliated Zhangye People's Hospital, Zhangye, Gansu, China; b Department of Endoscopy Center, Hexi University Affiliated Zhangye People's Hospital, Zhangye, Gansu, China; c Department of Image Center, Hexi University Affiliated Zhangye People's Hospital, Gansu, China; d Department of Pathology, Hexi University Affiliated Zhangye People's Hospital, Gansu, China; e Department of Urology, Institute of Urology, Hexi University, Zhangye, Gansu, China.

**Keywords:** liposarcoma, omentum, operative therapy

## Abstract

**Rationale:**

Omental liposarcoma is extremely rare, and only a few reports have been published in the literature. Due to the rarity of the disease, establishing a clear diagnosis and formulating a treatment plan may be challenging for clinicians.

**Patient concerns:**

The patient was a 51-year-old woman who presented with a protruding mass and pain in the lower abdomen.

**Diagnosis:**

Magnetic resonance imaging revealed a tumor measuring 15 cm in diameter in the pelvis. Ovarian cancer was suspected based on pre-operative imaging findings.

**Interventions:**

An exploratory laparotomy was performed. Intra-operative analysis of the frozen section suggested a benign tumor.

**Outcomes:**

Postoperative histopathological analysis confirmed the diagnosis of omental liposarcoma. The patient recovered well after surgery.

**Lesson:**

This case report helps clinical oncologists to develop a comprehensive understanding of this disease and treat it accordingly.

## 1 Introduction

The omentum is a double-layered membrane composed of the peritoneum and fatty tissue that contains blood vessels, nerves, lymphatic vessels, and connective tissue.^[1]^ The omentum is attached to the greater curvature of the stomach and transverse colon, covering the organs in the abdominal cavity and is shaped like a skirt. If infections, tumors, or other diseases occur in the abdominal cavity, the omentum can limit the spread of the disease through the formation of wraps and adhesions.^[2]^ The omentum is a common site for metastasis of ovarian, stomach, and colon cancer, and is also a common site of granulomatous inflammation, including tuberculous infection and fibrosis. Primary omental tumors are rare.^[3,4]^ According to the 2020 World Health Organization soft tissue tumor classification, most sarcomas originating in the omentum are soft tissue tumors.^[5]^ Soft tissue sarcoma is a rare malignant tumor derived from mesenchymal tissue cells that can occur in any part of the body.^[6]^ Liposarcoma and leiomyosarcoma are the most common soft tissue sarcomas in the abdominal cavity, and the retroperitoneum is the most common site. Omental sarcoma is very rare and can easily be misdiagnosed before surgery.^[7]^ Here, we present the case of a patient with omental liposarcoma, so that other clinical oncologists can develop a comprehensive understanding of this disease and treat it accordingly.

## 2 Case report

**Figure 1:**
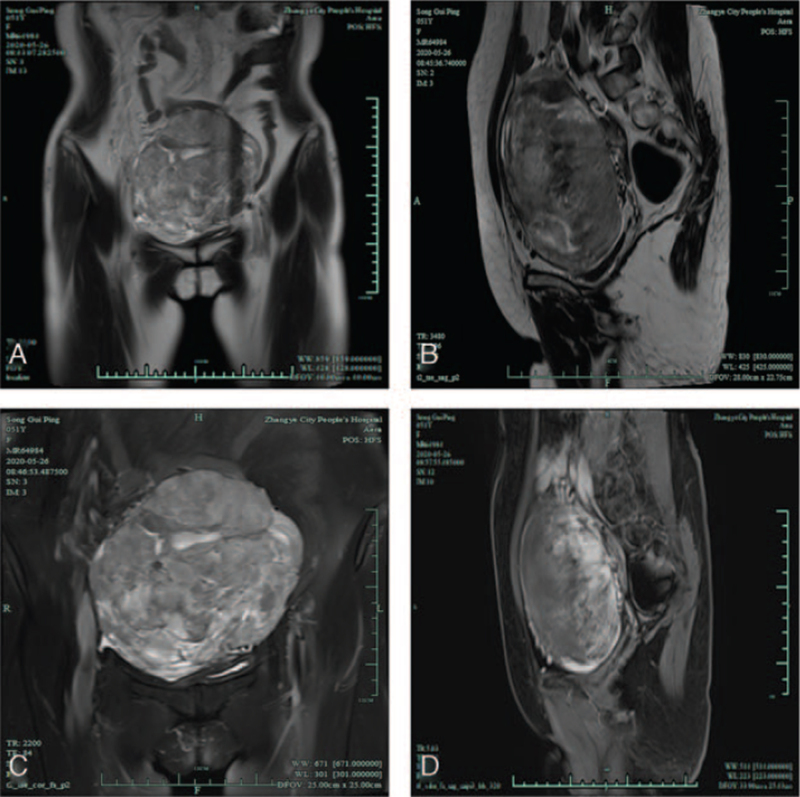
Magnetic resonance imaging of the pelvic cavity mass: (A) Coronal section; (B) Sagittal section; (C) Contrast-enhanced image of the coronal section; (D) Contrast-enhanced image of the sagittal section.

A 51-year-old female patient was admitted to the hospital with a protruding lower abdominal mass and sudden-onset lower abdominal pain with frequent urination. The patient started noticing a mass protruding from the lower abdominal wall 2 days before initial presentation; however, she did not pay attention to it at the time. The next day, she started experiencing sudden-onset, severe, and continuous lower abdominal pain after 2 hours of activity, which was accompanied by frequent urination. Abdominal ultrasonography performed at a local hospital revealed a large heterogeneous echo in the pelvic cavity. Ovarian tumor or uterine fibroids were suspected, and the patient was hospitalized for further examination and treatment. However, the patient did not seek further medical assistance after symptom relief. Two months later, the patient experienced abdominal pain again and was admitted to our hospital. Physical examination on admission revealed a body temperature of 36.6°C, blood pressure of 100/65 mm Hg, and respiratory rate of 20 breaths/min. On abdominal examination, a poorly mobile solid mass, approximately 15 cm in diameter, was palpable in the lower abdomen. Gynecological examination revealed a smooth mucous membrane of the vaginal canal, a normal cervix, and a large hard mass in the pelvic cavity with poor mobility and mild tenderness. Contrast-enhanced pelvic magnetic resonance imaging (MRI) showed a large mass in the pelvic cavity, which was considered an ovarian tumor. A small amount of fluid was present in the pelvis (Fig. 1).

**Figure 2:**
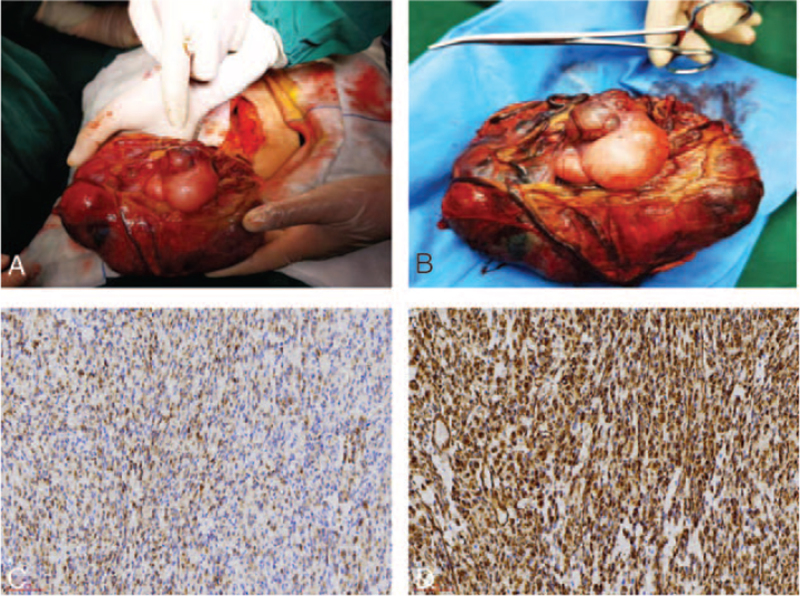
Gross and microscopic appearance of the tumor: (A) Intra-operative removal of omental space-occupying lesion; (B) Gross appearance of the omental liposarcoma specimen; Omental liposarcoma specimen visualized using immunohistochemical staining (C) Desmin positive staining ×200; (D) Vimentin positive staining ×200.

After discussing these findings with the patient and her family members, informed consent was obtained from the patient for exploratory laparotomy. After sufficient pre-operative preparation, laparotomy was performed under general anesthesia. During the operation, part of the greater omentum was found to be adhered to the anterior wall of the peritoneum and bladder, 200 mL of clear ascitic fluid was present in the abdominal cavity, and an isolated mass measuring approximately 15 cm in diameter protruding from the omental surface. The mass had a soft texture, was bluish-purple in color in some parts, appeared to contain different tissue types, and received abundant blood supply from the omentum. The base of the tumor adhered slightly to the right fallopian tube. There was no obvious abnormality in the appearance of the bilateral fallopian tubes and ovaries. General surgeons and urologists were consulted during the operation, and the mass from the omentum was considered malignant. Therefore, tumor and partial omental resection and pelvic adhesion lysis were performed (Fig. 2A, B). Histopathological analysis of the intra-operative frozen section showed an encapsulated grayish-brown omental mass that adhered to 21 cm of the omentum. Ten lymph nodes with a diameter of approximately 0.2 to 0.6 cm were also palpable. Based on the analysis of the intra-operative frozen section, the mass was considered a benign pelvic tumor. A drainage tube was placed in the abdominal cavity, and the abdomen was closed routinely. The patient recovered well after the operation.

On gross examination, the cut surface of the tumor was grayish-white to grayish-brown in color and had a consistency similar to that of fish meat. Patchy areas of necrosis were observed. On histological examination, the tumor was confirmed to be an omental liposarcoma (Fig. 2C, D). Immunohistochemical analysis was as follows: Cytokeratin (2+), CD99 (–), Ki-67 (10%), S-100 (–), Vimentin (3+), Desmin (2+), CD68 (–), CA125 (–), Epithelial membrane antigen (–), Smooth muscle actin (–), CD117 (–), CD34 (–), Dog 1 (–), CD10 (–). The patient was administered polyethylene glycol liposomal doxorubicin 80 mg intravenous D1 for a total of 4 cycles. After the initial treatment, the tumor was evaluated for complete response, and the patient was followed up every 3 months. At 18 months after the operation, the imaging examination results and tumor markers CA125 and alpha-fetoprotein were normal. Further follow-up is ongoing.

## 3 Discussion

Liposarcoma originates from primitive mesenchymal cells and is a common soft tissue malignancy.^[8]^ Liposarcoma is composed of lipoblasts and often occurs in fatty tissues present in the limbs, abdomen, and retroperitoneum. It is rare in the peritoneal cavity. Liposarcoma is usually a large, deep, painless, and gradually growing mass, and the course of the disease can often span several decades.^[9]^ The main clinical manifestations of omental liposarcoma are progressive abdominal distension, abdominal discomfort and pain, pantothenic acid deficiency, belching, and other gastrointestinal symptoms.^[10]^ Malignant tumors of the omentum include primary and secondary types. Primary tumors originate in the omentum and are mostly sarcomas or mesotheliomas. Secondary tumors originate in other organs, but later metastasize to the greater omentum.^[11,12]^ Omental liposarcoma is rarely found in clinical practice or autopsy. It is a slow-growing malignant tumor. It mostly occurs in men, and the incidence is more common around the age of 50 years. However, the patient in the present case was a female. Only a few cases have been reported in China and other countries. Secondary malignant tumors of the omentum are clinically common, but primary malignant tumors of the greater omentum are extremely rare. Ultrasonographic features are atypical and can be easily missed. Primary omental malignant tumors that have been reported include leiomyosarcoma, fibrosarcoma, liposarcoma, mesothelioma, and malignant fibrous histiocytoma.^[13]^ Secondary omental malignant tumors mostly originate from the metastasis of malignant tumors of the stomach, colon, pancreas, biliary tract, ovary, liver, etc, and the tumor size may vary.^[14,15]^ Currently, there is no specific diagnostic method for omental tumors. Ultrasonography has the advantages of being non-ionizing, simple, and repeatable. It can help identify lesions and locate them qualitatively. A pathological diagnosis can be obtained by combining an ultrasound-guided percutaneous biopsy. In addition, computed tomography (CT) helps in establishing the diagnosis and determining the treatment plan.^[16,17]^ However, since there is a wide variety of omental tumors that have different properties and at different stages of malignancy, the interpretation of ultrasonographic findings may be affected by the presence of intestinal gas and sound energy attenuation, which complicates the diagnosis of omental tumors. Therefore, when omental tumors are suspected, ultrasonography must be performed carefully. Moreover, an operator's cumulative experience and optimization of scanning technology may also increase the detection rate of omental tumors and reduce the number of missed diagnoses. Ultrasound has a certain value in determining the location and internal structure of omental malignant tumors. To determine the tumor properties, it is necessary to combine ultrasonography with clinical examination and other imaging modalities, such as CT and MRI.^[18]^ Surgical resection is the treatment of choice for omental liposarcomas. Depending on the stage of malignancy, local palliative resection, local extensive resection, or radical resection can be performed.^[19]^ For patients who cannot undergo radical surgical resection, palliative surgery, and postoperative radiotherapy and chemotherapy can often reduce the tumor recurrence rate and prolong survival. In the present case, radical resection of the tumor was performed. Partial resection of the corresponding organs should also be performed if the tumor infiltrates or adheres to the abdominal and pelvic organs.

The surgical procedure usually combines complete resection of the tumor and partial resection of the involved organs. Tumor size and histological subtype are important prognostic factors. Based on the pathology, the prognosis of omental liposarcoma is better. Since most patients present late, when only incomplete surgical resection can be performed, the recurrence rate is high. Most cases of recurrence occur at the original site, and another operation is generally required to remove the recurrence. The literature reports that there is no significant difference in the prognosis between 2 resections and more than 4 resections; therefore, repeat surgical resections can be performed. The patient underwent contrast-enhanced abdominal MRI before the operation. Ovarian tumor and pelvic effusion were suspected based on the MRI findings. Since the origin and properties of the tumor cannot be accurately determined on imaging, pre-operative diagnosis is difficult. Because omental liposarcoma is rare, there is a lack of clinical experience and related research, which makes establishing the diagnosis based on imaging even more complicated. In addition, the inability of pelvic MRI to identify the blood supply to the tumor and the failure to perform percutaneous biopsy before surgery also led to misdiagnosis. Moreover, intra-operative frozen section analysis suggested a benign tumor, and the diagnosis of omental sarcoma was established only after pathological analysis of the resected tumor. General surgeons and urologists were consulted to formulate the postoperative treatment plan, which further highlights the difficulty in diagnosing and treating omental sarcomas. Because there is no standard treatment plan, the clinician should summarize and discuss the plan with the patient, and continuous follow-up should be performed to observe the clinical features and prognosis of this tumor.

## 4 Conclusions

In summary, it is difficult to make a clear pre-operative diagnosis of omental liposarcoma, and most cases are diagnosed postoperatively. Currently, there are no reliable investigation modalities or specific markers to diagnose this tumor in clinical practice. Although B-ultrasound, CT, and MRI are the main imaging modalities, due to subjective and objective reasons, the misdiagnosis rate is high, which should garner our attention. Before the operation, a detailed medical history should be obtained, and a careful physical examination and necessary auxiliary investigations should be performed to improve the diagnosis rate. If a pre-operative diagnosis cannot be established, laparotomy should be performed. Due to the delay in treatment and incomplete surgical resection, the recurrence rate is relatively high, which worsens the prognosis. Therefore, early detection and initiation of treatment and thorough resection of the tumor are instrumental in improving the prognosis of omental liposarcoma.

## Acknowledgments

We would like to thank Editage (www.editage.cn) for English language editing.

## Author contributions

**Conceptualization:** Ying Gao, Yujie Qin, Xiaoyi Wei.

**Data curation:** Ying Gao, Yujie Qin, Yingchao Wang, Xiaoling Quan, Jiaxi Yao.

**Funding acquisition:** Ying Gao, Jiaxi Yao.

**Investigation:** Yujie Qin.

**Resources:** Yingchao Wang, Xiaoling Quan, Xiaoyi Wei.

**Writing – original draft:** Ying Gao, Yujie Qin.

**Writing – review & editing:** Xiaoyi Wei, Jiaxi Yao.
